# Opportunistic Osteoporosis Screening from Routine Knee Radiographs Using a Multi-Stage CNN Framework with External Validation

**DOI:** 10.3390/jcm15135222

**Published:** 2026-07-03

**Authors:** Nitiphoom Sinnathakorn, Chanon Fahpinyo, Watcharaporn Cholamjiak, Suthep Suantai

**Affiliations:** 1 School of Medicine, University of Phayao, Phayao 56000, Thailand; nitiphoom.si@up.ac.th (N.S.); chanon.fa@up.ac.th (C.F.); 2 School of Science, University of Phayao, Phayao 56000, Thailand; watcharaporn.ch@up.ac.th; 3 MedUP Intelligence Healthcare and Data Innovation Center, School of Medicine, University of Phayao, Phayao 56000, Thailand; 4 Research Center in Optimization and Computational Intelligence for Big Data Prediction, Department of Mathematics, Faculty of Science, Chiang Mai University, Chiang Mai 50200, Thailand

**Keywords:** osteoporosis, knee X-ray, CNN framework, feature extraction, external validation

## Abstract

**Background/Objectives:** Osteoporosis is a major public health concern associated with increased fracture risk and reduced quality of life if not detected at an early stage. Automated analysis of knee X-ray images using artificial intelligence has shown promising potential for opportunistic osteoporosis screening. This study aims to develop and evaluate a multi-stage deep learning and machine learning framework for osteoporosis classification, with particular emphasis on external validation, calibration drift, and cross-domain generalization performance. **Methods:** Knee X-ray images were categorized into three classes: Normal, Osteopenia, and Osteoporosis. Deep features were extracted using pretrained convolutional neural networks, including ResNet18, EfficientNetB0, and DenseNet121. The extracted features were subsequently classified using multiple machine learning models, including Neural Network, Efficient Linear, Support Vector Machine, and Naive Bayes classifiers. Two data augmentation strategies were investigated: targeted minority-class augmentation and full 3× dataset expansion with class balancing. Model performance was evaluated using accuracy, precision, recall, F1-score, and AUC on internal validation, independent test sets, and external validation datasets. Additional analyses included reliability calibration assessment, isotonic recalibration, and class-prior boosting with cross-validated threshold optimization to address external domain shift. **Results:** EfficientNetB0 and DenseNet121 consistently outperformed ResNet18 across most evaluation metrics. Under the balanced augmentation strategy, EfficientNetB0 combined with Efficient Linear demonstrated strong and stable performance, while DenseNet121 paired with a Neural Network achieved the highest overall classification performance. External validation revealed a substantial discrepancy between AUC and threshold-based metrics, indicating the presence of calibration drift and class-prior mismatch across imaging domains. Reliability analysis showed severe probability collapse in the Osteopenia class during external testing. Post-hoc recalibration improved probability reliability, while class-prior boosting substantially increased Osteopenia sensitivity and improved balanced accuracy and macro F1-score under external validation conditions. **Conclusions:** The proposed framework demonstrates the feasibility of combining pretrained CNN-based deep feature extraction with machine learning classifiers for osteoporosis classification from knee X-ray images. The findings further highlight that maintaining model performance under external testing conditions may require not only strong feature extraction capability but also adaptive recalibration and deployment-aware threshold optimization to address calibration drift and cross-domain variability. While the results are encouraging, the present study should be considered a proof-of-concept investigation. Although the framework was evaluated using an independent public external dataset, further validation using larger and more diverse multi-center clinical cohorts is necessary to establish generalizability and clinical utility before routine clinical implementation can be considered.

## 1. Introduction

Osteoporosis is a chronic and systemic skeletal disorder characterized by decreased bone mass, microarchitectural deterioration, and increased bone fragility and fracture [1]. The disease is observed in over 200 million individuals worldwide and is a significant public health challenge with specific attention to post-menopausal women and older individuals [2]. The worldwide burden of osteoporosis is escalating rapidly as a consequence of the aging population, sedentary lifestyles, and nutritional insufficiencies [3]. Osteoporotic fractures—especially those in the hip, spine, and wrist—cause substantial injury to the body, decrease quality of life, and cause increased mortality [4].

The gold standard for diagnosing osteoporosis is dual-energy X-ray absorptiometry (DXA) which measures bone mineral density (BMD) and classifies normal, osteopenic, and osteoporotic patients according to T-score thresholds determined by the World Health Organization [5]. Nevertheless, DXA has its own limitations, despite diagnostic precision. It demands expensive, specialized equipment and trained operatives, and is not uniformly available, particularly in rural or resource-light healthcare contexts [6]. Furthermore, DXA is a unilateral measure of bone density and does not capture microstructural alterations and cortical thinning seen clinically on radiographs [7].

Given these limitations, there has been growing interest in using existing radiographs, which are already commonplace in clinical practice for various other purposes, for the opportunity of screening patients for osteoporosis. A variety of X-rays, such as knee, hip, or spine, can indirectly indicate bone quality by the characteristics of texture, cortical thickness, and trabecular shape [8]. Knee radiographs are particularly frequently used in orthopedic and rheumatologic examinations which allow us to measure the condition of bone without the need for other radiation or additional expense [9]. The knee joint includes cortical and trabecular bone elements within the distal femur and proximal tibia areas that may reveal systemic bone changes related to the advancement of osteoporosis [10].

Medical image analysis has been revolutionized by the recent advancements of artificial intelligence (AI), especially deep learning (DL) and convolutional neural networks (CNNs). These models automatically capture hierarchical representations based on the imaging data, showing impressive capabilities in the diagnostic processes, segmentation, and disease classification across different medical fields [11]. For musculoskeletal imaging, CNNs have been used to estimate bone age, detect fractures, and pre-determine osteoporosis severity derived from radiographs or CT scans [12]. Multiple studies have shown that CNN-based systems can predict bone health status based on naked radiographs with similar or superior accuracy to that of conventional BMD classification techniques. For example, D’Agostino et al. (2024) has demonstrated high sensitivity and specificity in osteoporosis classification using deep learning on knee radiographs [13], and Gomathi et al. (2026) developed a hybrid deep-learning model, which revealed osteopenia as an intermediate stage, yielding significant accuracy gains [14]. Nonetheless, a significant number of these models were trained and validated on small datasets, often from only one institution, prompting concerns of overfitting and generalizability limitations [15]. To further enhance robustness and make the models more applicable into clinical settings, multi-stage CNN frameworks have also been presented. These models extract features and perform classification in a sequential or hierarchical manner and classification decisions processed in sequential or hierarchical manner to model the expert human reasoning [16]. Moreover, different CNN layers, architectures, and even image data modalities can be considered as complementary visual inputs, and feature fusion can offer a good option where many complementary visual cues can be merged together [17].

Multi-stage architectures combined with feature fusion can alleviate data imbalance and improve the learning of discriminative texture-based features from medical images [18]. The methodological novelty of the present study lies in the integration of a translational multi-stage architecture that combines complementary deep feature representations, targeted data augmentation, machine-learning-based feature learning, and independent external validation within a unified osteoporosis screening framework. Unlike conventional end-to-end CNN models that rely on a single feature extractor, the proposed framework systematically investigates multiple CNN architectures and exploits feature fusion to capture complementary cortical and trabecular bone characteristics. This design aims to improve feature diversity, enhance robustness against imaging variability, and provide a framework with improved potential for clinical translation and external applicability. However, even considering the methodological improvements, an important step often missing from AI research is external validation. Models designed and trained internally often model fine results but fail to apply to a diverse set of data (across institutions, imaging systems, and patient populations) [19]. External validation assesses model generalizability and real-world performance, critical for both clinical translation and regulatory acceptancy [20,21]. As a result, including real-world (hospital) data as an external test set offers stronger evidence of the clinical robustness and applicability.

When the patients are involved for their osteoporosis, external validation is of exceptional importance due to the heterogeneity, the variations in imaging characteristics, and the differences in bone characteristics between ethnic groups [22]. For instance, from the Kaggle Osteoporosis Knee Dataset, quality, de-identified radiographs are provided, divided into the normal OTS, osteopenia, and osteoporosis groups [23]. This dataset is perfect for the model building and pilot internal validation. Nevertheless, clinical validity needs validation on hospital-based datasets [24], which includes University of Phayao Hospital, with Normal = 41, Osteopenia = 34, and Osteoporosis = 41, to ensure the model validates across varied imaging modalities and populations [24]. The project, “Translational Multi-Stage CNN Framework with Feature Fusion and External Validation to classify Osteoporosis and Knee X-rays”, focuses on bridging the gap between algorithmic progress and clinical implementation. By using deep learning with feature fusion and validation based on the performance alone, the work adheres to the principles of translational medicine that can convert computational techniques into diagnostic tools that assist in early diagnosis and clinical decision [25].

The intended clinical use of the proposed framework is as an opportunistic osteoporosis screening tool integrated into routine radiographic workflows. In many healthcare settings, knee radiographs are frequently acquired for patients with osteoarthritis, knee pain, or musculoskeletal complaints. The proposed model can automatically analyze these existing images and identify individuals at increased risk of osteopenia or osteoporosis without requiring additional imaging examinations. Patients classified as high risk can subsequently be referred for confirmatory DXA assessment and specialist evaluation. This workflow has the potential to support opportunistic osteoporosis screening and clinical decision-making, particularly in resource-limited settings where DXA availability is restricted.

Finally, this research aims at proposing a clinically significant AI-assisted screening framework for deployment in hospitals with few DXA scanners. This could facilitate earlier identification of individuals at risk of osteoporosis and support preventive healthcare strategies for aging populations [26]. Furthermore, the proposed framework contributes beyond osteoporosis classification by demonstrating how multi-stage deep learning, feature fusion, and rigorous external validation can be integrated within a translational AI pipeline. The framework is designed not only to maximize predictive performance but also to enhance robustness, generalizability, and clinical applicability, which are essential requirements for the deployment of AI systems in real-world healthcare environments.

## 2. Materials and Methods

### 2.1. Dataset Description

**Figure 1:**
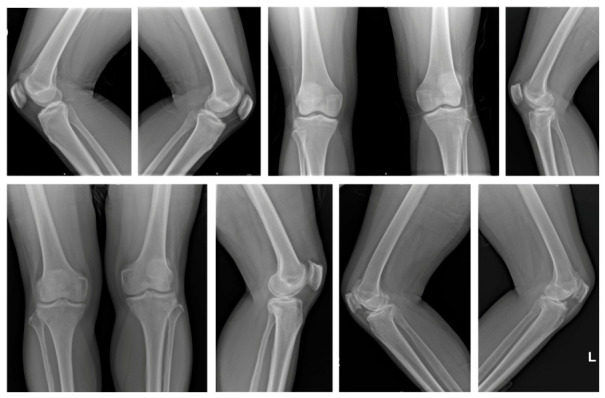
Representative samples of knee X-ray images from the MedUP Intelligence Healthcare and Data Innovation Center, School of Medicine, University of Phayao. The images demonstrate variability in anatomical structures, imaging orientations (anterior–posterior and lateral views), and bone density patterns across the three classes: Normal, Osteopenia, and Osteoporosis. These variations highlight the complexity of the dataset and the necessity for robust feature extraction models.

The data used in this study were retrieved from the University Phayao Hospital, Thailand, from knee X-ray images of patients classified into three categories in Normal, Osteopenia, and Osteoporosis. The dataset consisted of 116 patients, including 41 Normal, 34 Osteopenia, and 41 Osteoporosis cases. Multiple radiographs were available for some patients, resulting in a total of 322 knee X-ray images (119 Normal, 90 Osteopenia, and 113 Osteoporosis). Dataset partitioning was performed at the patient level to ensure that no patient appeared in more than one subset. The class distribution is shown in Figure 1. The data were analyzed and the patients’ diagnosis made by medical representatives was used. We then split the dataset into training, validation, and testing sets within the group and in a hierarchical fashion-maintained class distribution. The data were split directly before any augmentation steps are completed due to risk of data leaking, in order for us to verify our model’s generalizability. To verify the model’s generalizability, we used an external validation dataset. We selected 150 knee X-ray images on Kaggle (50 in each class) and then randomly distributed the images. This external dataset was not used for training or model selection, and all test data were drawn from this dataset. Medical data were analyzed to meet ethical criteria and all patient data in accordance with institutional and data protection regulations.

From Figure 1, representative knee X-ray images from the MedUP Intelligence Healthcare and Data Innovation Center, School of Medicine, University of Phayao illustrate substantial variability in anatomical structures, imaging orientations (anterior–posterior and lateral views), and bone density patterns across the Normal, Osteopenia, and Osteoporosis classes. However, the visual differences between classes—particularly between Osteopenia and Osteoporosis—are subtle and often overlapping, making reliable classification by human observation alone challenging. This highlights the limitation of visual assessment and underscores the need for automated deep feature extraction and machine learning approaches to achieve more accurate and consistent classification. Bone mineral density (BMD) measurements obtained by dual-energy X-ray absorptiometry (DXA) were used as the reference standard for osteoporosis classification. Diagnostic categories were assigned according to the World Health Organization (WHO) criteria based on T-scores. Subjects with T-scores greater than or equal to −1.0 were classified as Normal, subjects with T-scores between −1.0 and −2.5 were classified as Osteopenia, and subjects with T-scores less than or equal to −2.5 were classified as Osteoporosis. The corresponding DXA examinations were obtained as part of routine clinical care, and the diagnostic labels were extracted from the medical records.

### 2.2. Data Preprocessing and Augmentation

All knee X-ray images were preprocessed to ensure consistency and compatibility with deep learning models. First, all images were resized to a fixed resolution of 224 × 224 pixels. Since the original images were in grayscale format, they were converted into three-channel images by replicating intensity values across RGB channels.

The dataset was divided into training, validation, and testing subsets using a stratified sampling strategy to preserve class distribution. Importantly, the data splitting process was performed prior to augmentation to prevent data leakage and ensure unbiased evaluation.

To enhance model generalization and mitigate class imbalance, two augmentation strategies were employed exclusively on the training set:

1. Targeted augmentation:
  Data augmentation was selectively applied with a greater emphasis on the Osteopenia and Osteoporosis classes compared to the Normal class. This approach aims to enhance the representation of clinically relevant conditions while mitigating class imbalance in the training dataset.

2. Balanced 3× augmentation:
  All training samples were augmented to achieve approximately threefold expansion, followed by a class balancing process. In this step, each class was oversampled to match the size of the largest class, resulting in a balanced training dataset.

Data augmentation was applied only to the training set after patient-level data splitting. The augmentation function included both geometric and intensity-based transformations designed to simulate realistic variability in knee X-ray acquisition. Geometric transformations consisted of random rotation within ±12°, random scaling between 0.90 and 1.10, translation up to approximately 8% of the image size, mild shear transformation within ±5°, and random horizontal flipping with a probability of 0.5. Intensity-based augmentation included random brightness adjustment within ±0.10, contrast scaling within ±15%, gamma correction in the range of 0.90–1.10, additive Gaussian noise with σ between 0.003 and 0.013, and mild Gaussian blurring with σ between 0.3 and 1.0. All transformed images were clipped to the intensity range [0, 1], resized to the required CNN input size and converted to three-channel RGB format.

All augmentations were implemented using an on-the-fly transformation pipeline, allowing augmented images to be generated dynamically during training without permanently modifying the original dataset. This approach improves computational efficiency and avoids unnecessary data duplication.

From Table 1, the dataset initially contained 322 knee radiographs and was split into 257 training and 65 testing samples. The train–test partition was performed at the patient level to eliminate the risk of information leakage, ensuring that no patient appeared in more than one subset. Therefore, the training and testing sets were completely independent and consisted of images from different patients. To mitigate class imbalance, minority augmentation and full 3× augmentation were applied, followed by class balancing, resulting in a final training set of 855 images with equal class distribution, while the test set remained unchanged.

### 2.3. Deep Feature Extraction Models

Deep feature extraction plays a crucial role in medical image analysis, particularly for capturing complex patterns associated with bone density variations in knee X-ray images. In this study, pretrained convolutional neural networks (CNNs) were employed as feature extractors to transform input images into high-level representative feature vectors. Instead of training deep networks from scratch, transfer learning was utilized to leverage models pretrained on the ImageNet dataset, which has been shown to improve performance and convergence in medical imaging tasks.

Three state-of-the-art CNN architectures were selected, namely ResNet18 [16], EfficientNetB0 [17], and DenseNet-121 [27]. These models were chosen due to their complementary design principles and proven effectiveness in image representation learning. Specifically, ResNet18 utilizes residual connections to facilitate gradient flow, EfficientNetB0 adopts compound scaling with mobile inverted bottleneck (MBConv) blocks for efficient feature extraction, and DenseNet-121 employs dense connectivity to enhance feature reuse and information propagation.

For each model, feature extraction was performed by removing the final classification layer and obtaining features from the global pooling layer. The extracted feature dimensions were 512 for ResNet18, 1280 for EfficientNetB0, and 1920 for DenseNet-121. These feature vectors were subsequently used as input for machine learning classifiers in the next stage.

**Figure 2:**
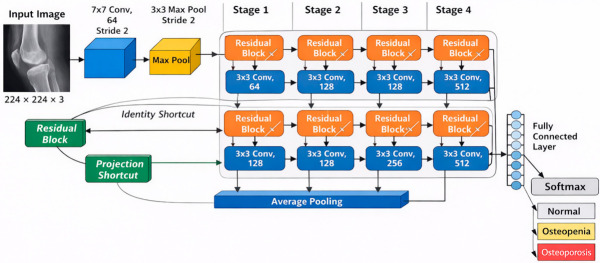
ResNet18 architecture for knee X-ray-based osteoporosis classification, consisting of convolution, residual blocks, global average pooling, and a fully connected layer, with softmax outputs for Normal, Osteopenia, and Osteoporosis.

**Figure 3:**
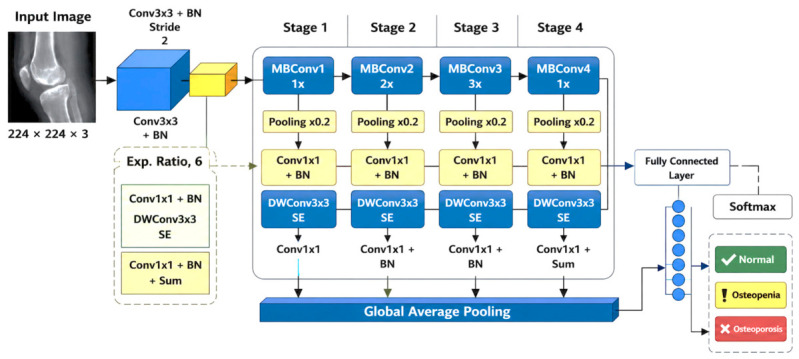
EfficientNet-B0 architecture for knee X-ray-based osteoporosis classification, utilizing MBConv blocks with squeeze-and-excitation, global average pooling, and a fully connected layer with softmax outputs for Normal, Osteopenia, and Osteoporosis.

**Figure 4:**
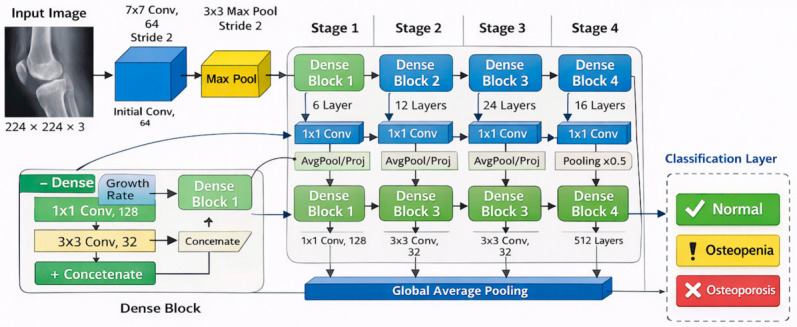
DenseNet-121 architecture for knee X-ray-based osteoporosis classification, leveraging dense blocks with feature concatenation, global average pooling, and a classification layer with softmax outputs for Normal, Osteopenia, and Osteoporosis.

To comprehensively extract discriminative features from knee X-ray images, three state-of-the-art convolutional neural network (CNN) architectures, namely ResNet18, EfficientNet-B0, and DenseNet-121 (Figure 2, Figure 3 and Figure 4), were employed. As summarized in Table 2, these models differ in terms of architectural design, feature dimensionality, and extraction mechanisms. Specifically, ResNet18 utilizes residual connections to mitigate the vanishing gradient problem and enable stable deep feature learning. EfficientNet-B0 adopts compound scaling with MBConv blocks and squeeze-and-excitation modules to achieve an optimal balance between efficiency and performance. DenseNet-121 leverages dense connectivity to promote feature reuse and strengthen gradient propagation across layers. These complementary characteristics provide diverse and informative feature representations, thereby enhancing the robustness and generalization capability of the proposed osteoporosis classification framework.

All knee radiographs were resized to the input resolution required by each pretrained CNN architecture and converted to three-channel images by replicating the original grayscale image across the RGB channels. Pixel intensities were normalized to the range [0, 1] using MATLAB (R2025a) image preprocessing functions. The pretrained ResNet18, EfficientNet-B0, and DenseNet121 models were employed solely as fixed feature extractors, and no fine-tuning was performed. Consequently, no learning-rate scheduling, batch-size optimization, epoch-based training, early stopping, or weight updating procedures were required. Deep features extracted from the final feature layers of the pretrained networks were subsequently used as inputs for the machine learning classifiers implemented in MATLAB Classification Learner. Feature extraction was performed after all preprocessing and augmentation procedures were completed within the training set, ensuring that no information from the independent test or external validation datasets was used during model development.

From Table 2, three pretrained convolutional neural networks, namely ResNet18, EfficientNetB0, and DenseNet-121, were employed as deep feature extractors. Features were extracted from the global pooling layer of each network, specifically pool5 for ResNet18, global_average_pooling2d_1 for EfficientNetB0, and avg_pool for DenseNet-121. The extracted feature dimensions were 512, 1280, and 1920, respectively. ResNet18 provides compact and computationally efficient representations, EfficientNetB0 offers a balanced trade-off between efficiency and discriminative power, while DenseNet-121 yields the richest feature representation due to its densely connected architecture.

### 2.4. Machine Learning Classifiers

To classify the deep features extracted from knee X-ray images, five optimizable machine learning classifiers implemented in MATLAB Classification Learner (Version R2025a) [28] were employed: Optimizable Tree [29], Optimizable SVM [30], Optimizable Naive Bayes [31], Optimizable KNN [32], Optimizable Neural Network [33], and Optimizable Efficient Linear [34]. These classifiers were selected to represent different learning mechanisms, including rule-based partitioning, margin-based classification, probabilistic modeling, distance-based learning, and nonlinear neural mapping.

The Optimizable Tree classifier partitions the feature space hierarchically and is effective for modeling nonlinear decision boundaries. The Optimizable SVM identifies an optimal separating hyperplane and is particularly suitable for high-dimensional deep feature representations. The Optimizable Naive Bayes classifier provides a probabilistic framework with high computational efficiency, serving as a useful baseline model. The Optimizable KNN classifier predicts labels based on local neighborhood relationships in the feature space, making it suitable for evaluating local feature similarity. The Optimizable Neural Network classifier captures complex nonlinear interactions and provides flexible decision boundaries for multi-class classification. The Optimizable Efficient Linear classifier employs regularized linear decision functions and offers an efficient alternative for high-dimensional feature spaces.

All classifiers were implemented using MATLAB Classification Learner and trained using Bayesian hyperparameter optimization with five-fold cross-validation performed exclusively on the training set. The objective of the optimization procedure was to maximize cross-validation classification accuracy while identifying model-specific parameter settings. 

The optimized hyperparameters, search ranges, and final selected values for each classifier are summarized in Table 3. By evaluating these classifiers on the extracted deep features, this study aims to identify the most suitable machine learning model for classifying knee X-ray images into Normal, Osteopenia, and Osteoporosis categories.

### 2.5. Overall Workflow

**Figure 5:**
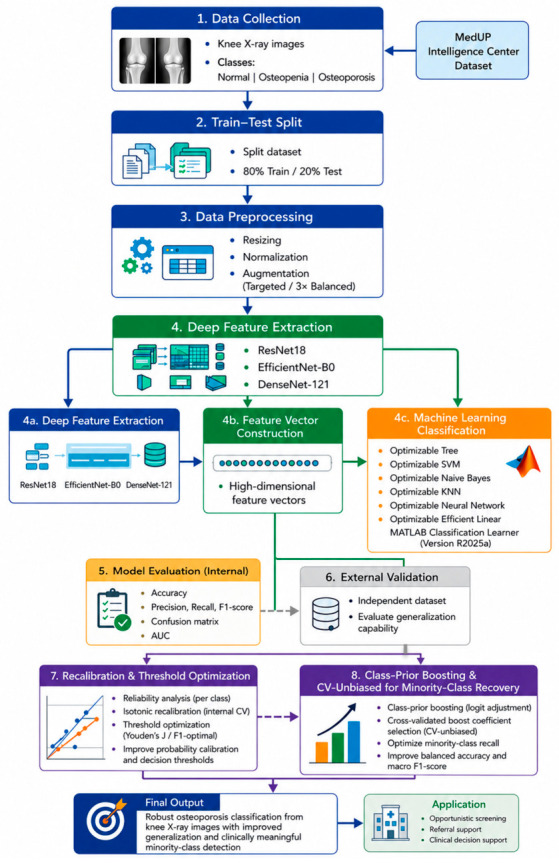
Overall workflow of the proposed osteoporosis classification framework. Knee X-ray images from the MedUP Intelligence Center dataset are first split into training and testing sets, followed by preprocessing. Deep features are extracted using ResNet18, EfficientNet-B0, and DenseNet-121, and subsequently classified using optimizable machine learning models in MATLAB Classification Learner. Model performance is evaluated, and external validation is conducted to assess generalization capability. Figure created by the authors using AI-assisted visualization tools (OpenAI ChatGPT (GPT-5.3) and DALL·E).

From Figure 5, the proposed workflow integrates deep learning, machine learning, and deployment-oriented calibration strategies for osteoporosis classification from knee X-ray images. The dataset is first divided into training and testing sets, followed by preprocessing steps including resizing, normalization, and augmentation. To improve model robustness and reduce potential bias from data partitioning, a 5-fold cross-validation strategy was employed during model development. All cross-validation procedures and classifier hyperparameter optimization were performed exclusively within the training set to prevent information leakage and ensure unbiased model selection. Deep feature extraction was subsequently performed using multiple pretrained CNN architectures to capture diverse and discriminative image representations. These extracted features were used to construct high-dimensional feature vectors, which were then classified using optimizable machine learning models. Model performance was evaluated using standard classification metrics, including accuracy, precision, recall, F1-score, confusion matrix, and AUC. Finally, an independent external validation dataset, which remained completely isolated throughout model development and optimization, was used only once for final performance assessment to evaluate model generalization under independent external imaging conditions. The external validation dataset was obtained from the publicly available Multi-Class Knee Osteoporosis X-ray Dataset on Kaggle. As the dataset was collected and curated by an independent source, detailed information regarding image acquisition protocols, imaging equipment, DXA procedures, image quality control, and label generation was not fully available. Consequently, the external dataset represents a substantially different imaging domain from the internal clinical dataset. Therefore, it should be interpreted as an independent public-domain dataset and a stress test of model generalizability rather than a fully controlled multi-center clinical validation cohort. Nevertheless, its use provides valuable insights into the effects of domain shift and the robustness of the proposed framework under heterogeneous external conditions.

To further address calibration drift and domain shift observed during external validation, an additional recalibration and threshold optimization stage is incorporated using reliability analysis, isotonic recalibration, and threshold adjustment strategies. Furthermore, a class-prior boosting and CV-unbiased minority-class recovery framework is introduced to mitigate class-prior mismatch and improve Osteopenia sensitivity under external deployment conditions. These additional stages enhance probability calibration, balanced accuracy, and minority-class detection performance, thereby improving the clinical applicability and robustness of the proposed framework in real-world screening scenarios.

## 3. Results

### 3.1. Targeted Augmentation (3× Augmentation)

This section presents the results obtained after applying data augmentation specifically to minority classes by expanding their samples threefold (3×). The objective of this approach is to mitigate class imbalance while preserving the original distribution of majority classes. Performance metrics including accuracy, precision, recall, and F1-score are reported to evaluate the effectiveness of minority class augmentation. In addition, confusion matrices are analyzed to assess improvements in classification performance, particularly for underrepresented classes such as Osteoporosis.

From Table 4, the highlighted results indicate that EfficientNet-B0 combined with Neural Network and Efficient Linear classifiers achieved consistently strong performance across both validation and test sets. In particular, the Neural Network model demonstrated high classification capability with a validation accuracy of 77.11 and a test accuracy of 73.85, along with balanced precision, recall, and F1-score. Similarly, the Efficient Linear classifier showed competitive performance with a validation accuracy of 77.80 and a test accuracy of 72.31, while also achieving the highest AUC (0.8887) among EfficientNet-B0-based models, indicating strong discriminative ability.

**Figure 6:**
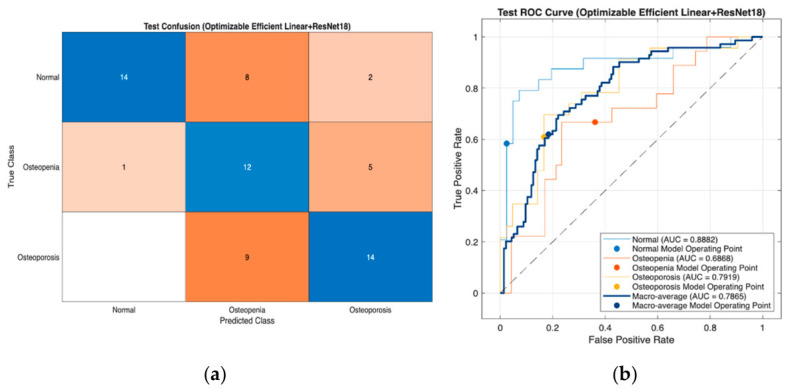
(**a**) Confusion matrix of the Optimizable Efficient Linear classifier using ResNet18 features on the test set, illustrating class-wise prediction performance for Normal, Osteopenia, and Osteoporosis. (**b**) Receiver operating characteristic (ROC) curves with corresponding AUC values for each class and macro-average, demonstrating the classification capability of the proposed model.

**Figure 7:**
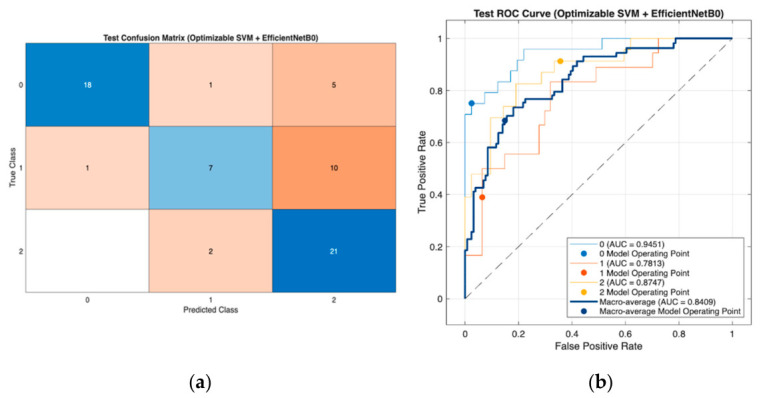
(**a**) Confusion matrix of the Optimizable SVM classifier using EfficientNet-B0 features on the test set, illustrating classification performance across Normal, Osteopenia, and Osteoporosis classes. (**b**) Receiver operating characteristic (ROC) curves with corresponding AUC values for each class and macro-average, demonstrating the discriminative performance of the model.

**Figure 8:**
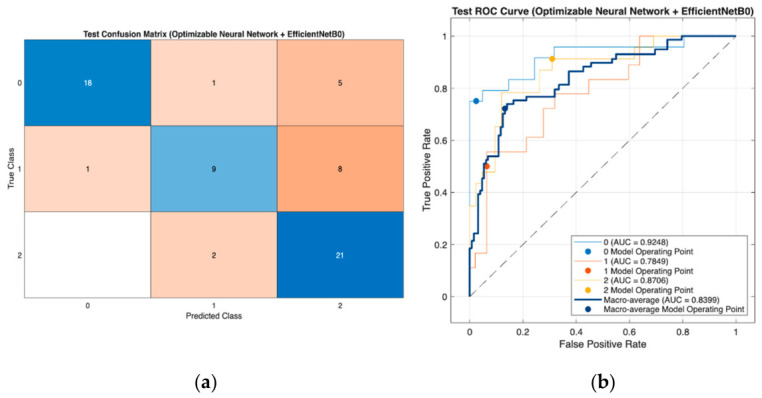
(**a**) Confusion matrix of the Optimizable Neural Network classifier using EfficientNet-B0 features on the test set, illustrating classification performance for Normal, Osteopenia, and Osteoporosis classes. (**b**) Receiver operating characteristic (ROC) curves with corresponding AUC values for each class and macro-average, demonstrating the model’s discriminative ability.

**Figure 9:**
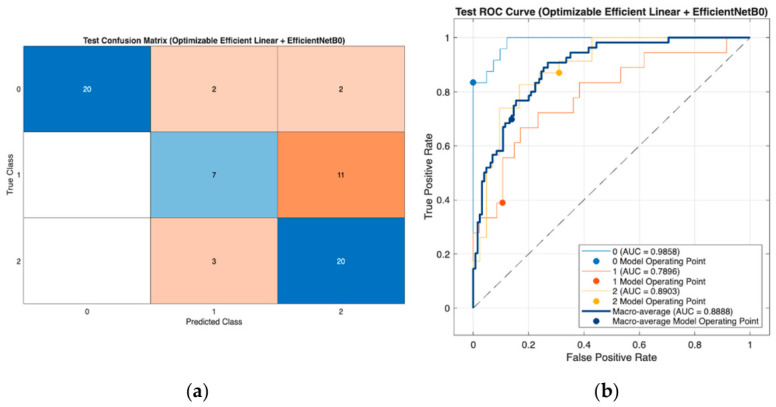
(**a**) Confusion matrix of the Optimizable Efficient Linear classifier using EfficientNet-B0 features on the test set, illustrating classification performance for Normal, Osteopenia, and Osteoporosis classes. (**b**) Receiver operating characteristic (ROC) curves with corresponding AUC values for each class and macro-average, demonstrating the effectiveness of the model in distinguishing between classes.

**Figure 10:**
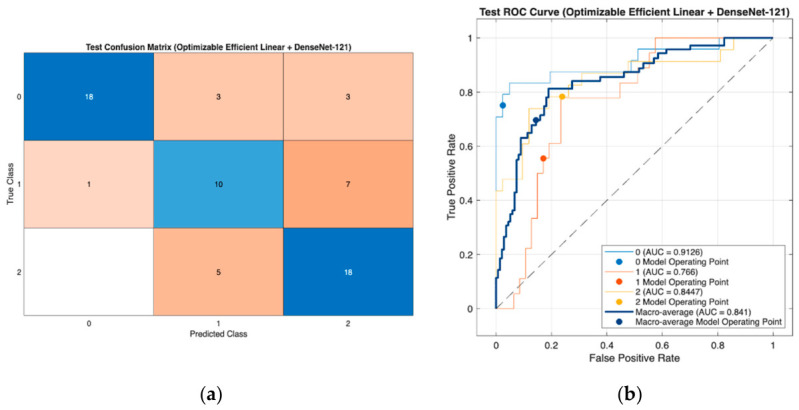
(**a**) Confusion matrix of the Optimizable Efficient Linear classifier using DenseNet-121 features on the test set, illustrating classification performance for Normal, Osteopenia, and Osteoporosis classes. (**b**) Receiver operating characteristic (ROC) curves with corresponding AUC values for each class and macro-average, demonstrating the discriminative performance of the model.

**Figure 11:**
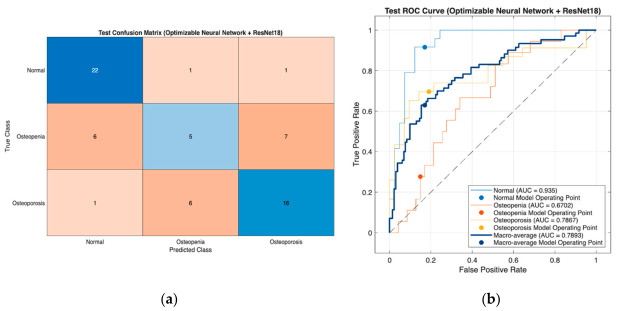
(**a**) Confusion matrix of the Optimizable Neural Network classifier using ResNet18 features on the test set, illustrating classification performance for Normal, Osteopenia, and Osteoporosis classes. (**b**) Receiver operating characteristic (ROC) curves with corresponding AUC values for each class and macro-average, demonstrating the model’s discriminative performance.

**Figure 12:**
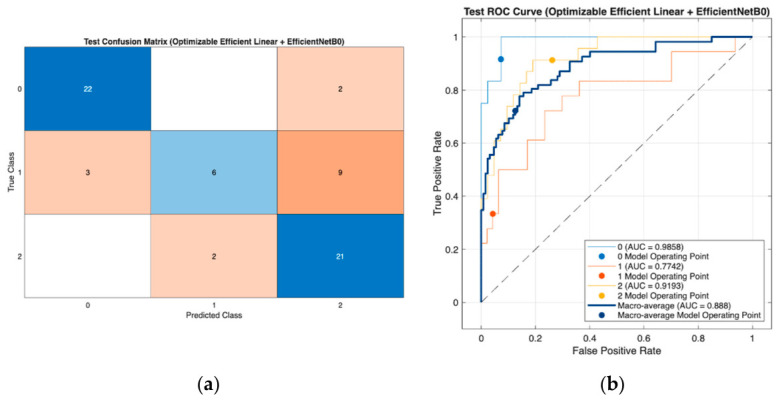
(**a**) Confusion matrix of the Optimizable Efficient Linear classifier using EfficientNet-B0 features on the test set, illustrating classification performance for Normal, Osteopenia, and Osteoporosis classes. (**b**) Receiver operating characteristic (ROC) curves with corresponding AUC values for each class and macro-average, demonstrating the model’s discriminative performance.

**Figure 13:**
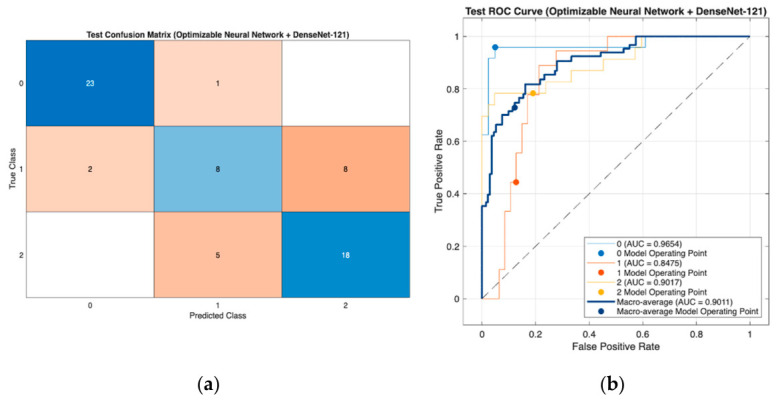
(**a**) Confusion matrix of the Optimizable Neural Network classifier using DenseNet121 features on the test set, illustrating classification performance for Normal, Osteopenia, and Osteoporosis classes. (**b**) Receiver operating characteristic (ROC) curves with corresponding AUC values for each class and macro-average, demonstrating the model’s discriminative performance.

Furthermore, DenseNet-121 combined with the Neural Network classifier achieved the best overall performance among all model combinations. This model obtained the highest validation accuracy (84.51) and strong test accuracy (67.69), along with the highest F1-score (66.02), reflecting its superior ability to learn highly informative feature representations. The results suggest that DenseNet-121 provides richer and more discriminative features, which are effectively leveraged by the Neural Network classifier to improve classification performance. To provide a more comprehensive interpretation of the classification performance, Figure 6, Figure 7, Figure 8, Figure 9, Figure 10, Figure 11, Figure 12 and Figure 13 present the confusion matrices and ROC curves for the representative model combinations evaluated on the independent test set. The test set consisted of 65 patient-level knee radiographs, including 24 Normal, 18 Osteopenia, and 23 Osteoporosis cases. Reporting the class-specific sample sizes facilitates a clearer assessment of sensitivity, specificity, and misclassification patterns across the three diagnostic categories.

From Figure 6, Figure 7, Figure 8, Figure 9 and Figure 10, the confusion matrices across all model configurations demonstrate that the proposed framework is capable of distinguishing between Normal, Osteopenia, and Osteoporosis classes with varying degrees of accuracy, depending on the feature extractor and classifier combination. In general, most models show strong performance in correctly identifying the Normal and Osteoporosis classes, while misclassification is more frequently observed in the Osteopenia class. This is expected due to the intermediate nature of Osteopenia, which shares overlapping characteristics with both Normal and Osteoporosis conditions.

Among all combinations, models based on EfficientNet-B0 and DenseNet-121 exhibit improved classification consistency, as reflected by higher numbers of correctly classified samples along the diagonal of the confusion matrices. In particular, the Efficient Linear and Neural Network classifiers demonstrate reduced misclassification rates, especially in distinguishing Osteoporosis cases, which is clinically important for screening applications.

The ROC curve analysis further supports these findings. Across all models, high AUC values are observed for the Normal class, indicating strong separability. The Osteoporosis class also shows relatively high AUC values, suggesting effective detection of severe cases. In contrast, the Osteopenia class consistently yields lower AUC values, reflecting the inherent difficulty in separating borderline cases. Macro-average AUC values across models generally fall within a good performance range, confirming the overall discriminative capability of the proposed framework.

Notably, the combination of EfficientNet-B0 with Efficient Linear achieves one of the highest AUC values, indicating excellent class separability. Similarly, DenseNet-121 combined with Neural Network provides strong and stable ROC performance, reinforcing its effectiveness in capturing complex feature representations.

From Table 5, the class-wise analysis revealed substantial differences in detection performance among the evaluated models. EfficientNetB0-based models consistently achieved the highest sensitivity for Osteoporosis (86.96–91.30%), indicating strong capability for identifying advanced bone loss. However, Osteopenia remained the most challenging class, with sensitivity ranging from 38.89% to 66.67%, reflecting the inherent difficulty of distinguishing intermediate disease stages. Among all models, DenseNet121 combined with Efficient Linear provided the most balanced performance across classes, achieving Osteopenia sensitivity of 55.56% while maintaining high specificity. These findings support the use of class-specific evaluation metrics in addition to overall accuracy and AUC, particularly when the intended clinical application is opportunistic screening, where minimizing missed Osteopenia and Osteoporosis cases may be more important than reducing false-positive referrals.

### 3.2. Full Dataset Expansion and Class Balancing (3× + Oversampling)

In this section, all classes are uniformly expanded by a factor of three (3×), followed by class balancing through oversampling to match the maximum class size. This strategy aims to enhance both data diversity and class balance simultaneously. The results are compared with those obtained from the minority-only augmentation strategy to examine the impact of full expansion and balancing on model performance. Special attention is given to changes in sensitivity and specificity across all classes, as well as overall model stability.

From Table 6, the results under the full dataset expansion and class balancing strategy demonstrate a clear improvement in model performance compared to the minority-only augmentation approach. Overall, EfficientNet-B0 and DenseNet-121 consistently outperform ResNet18, indicating that more advanced feature extractors provide more discriminative representations when sufficient and balanced data are available.

Among all combinations, EfficientNet-B0 with Efficient Linear achieves one of the best performances, with high test accuracy (75.38) and strong AUC (0.8880), highlighting its effectiveness and computational efficiency. Similarly, DenseNet-121 with Neural Network delivers the best overall performance, achieving the highest test accuracy (75.38) and F1-score (72.45), along with the highest AUC (0.9010), demonstrating superior generalization and feature learning capability.

In contrast, ResNet18-based models show comparatively lower performance, particularly on the test set, suggesting limited feature richness. Across classifiers, Neural Network and Efficient Linear consistently provide better generalization, while SVM performs well on validation but tends to show performance degradation on the test set. Naive Bayes remains the least effective despite significantly higher training time, indicating its limitations in handling high-dimensional deep features.

From Figure 11, Figure 12 and Figure 13, the results from the confusion matrices and ROC curves indicate that all models demonstrate strong performance in distinguishing Normal and Osteoporosis classes, while misclassification is more frequently observed in the Osteopenia class due to its intermediate characteristics.

Among the evaluated models, EfficientNetB0 combined with the Efficient Linear classifier achieves the best overall performance, particularly in terms of AUC and classification balance across classes. DenseNet121 with Neural Network also shows competitive performance, especially in feature representation, while ResNet18 provides comparatively lower but stable results.

Overall, EfficientNetB0 and DenseNet121 consistently outperformed ResNet18 across most evaluation metrics. DenseNet121 combined with Neural Network achieved the best overall test performance, whereas EfficientNetB0 with Efficient Linear provided the most stable balance between validation and test performance.

The ROC analysis further confirms these findings, with EfficientNetB0 achieving the highest macro-average AUC, followed by DenseNet121 and ResNet18. Overall, the results demonstrate that deeper and more advanced architectures yield more discriminative features for osteoporosis classification.

From Table 7, the 3× oversampling strategy substantially improved the detection of Normal and Osteoporosis cases, with DenseNet121 + Neural Network achieving the highest Normal sensitivity (95.83%) and EfficientNetB0 + Efficient Linear obtaining the highest Osteoporosis sensitivity (91.30%). However, Osteopenia remained the most challenging class across all models, with sensitivity ranging from 27.78% to 44.44%. Among the evaluated approaches, DenseNet121 + Neural Network provided the most balanced performance, achieving the highest Osteopenia sensitivity (44.44%) while maintaining high specificity across all classes. These results suggest that although oversampling improved overall discriminative performance, intermediate disease stages remained more difficult to identify than Normal and Osteoporosis cases.

### 3.3. External Validation Results

To assess the generalization capability of the proposed framework, external evaluation was conducted using an independent publicly available knee X-ray dataset. The trained models were evaluated without retraining to assess performance under external testing conditions and distributional shift. Performance metrics such as accuracy, precision, recall, and F1-score are reported, along with confusion matrices to analyze class-wise prediction behavior. The external evaluation provides additional evidence regarding the framework’s behavior under domain shift; however, the dataset should be interpreted as an external stress test rather than a formal multi-center clinical validation cohort.

From Table 8, the external validation results revealed a clear discrepancy between threshold-dependent metrics (accuracy, precision, recall, and F1-score) and threshold-independent discrimination performance measured by AUC. Although most models achieved relatively high AUC values (0.72–0.89), the corresponding external test accuracy remained substantially lower (31–44%), suggesting the presence of calibration drift and domain shift between the internal and external datasets.

This phenomenon indicates that the models preserved their ability to rank disease severity correctly, while the predicted probability distributions shifted under external conditions. Consequently, fixed decision thresholds such as argmax or 0.5 probability cutoffs became less reliable, leading to reduced classification accuracy despite acceptable AUC performance.

A major contributing factor is domain shift in knee X-ray images acquired from different institutions, including variations in imaging protocols, detector characteristics, image contrast, noise distribution, patient positioning, and demographic composition. These differences alter the feature distributions extracted by the CNN models, causing downstream classifiers trained on internal data to generalize poorly to external datasets.

The 3× oversampling strategy further supports this interpretation. While oversampling substantially improved AUC performance (0.87–0.89), external test accuracy did not improve consistently and occasionally decreased. This suggests that oversampling enhanced feature separability and minority-class ranking performance but also introduced posterior probability bias due to artificially balanced training distributions, resulting in poor threshold-based predictions on external data.

Additionally, the relatively small external test set size may have contributed to instability in accuracy and F1-score estimates. In multi-class classification, macro-average One-vs-Rest AUC can remain high even when argmax classification fails, particularly when confusion occurs between clinically adjacent categories such as Osteopenia and Osteoporosis.

Overall, these findings suggest that the proposed framework successfully learned clinically meaningful representations that generalize across datasets. However, the primary limitation lies in probability calibration and threshold transferability across imaging domains rather than in the feature extraction capability itself.

### 3.4. Recalibration and Threshold Optimization for External Deployment

To further investigate the discrepancy between AUC and threshold-based metrics during external validation, recalibration analysis was performed using the EfficientNet-B0 feature extractor with the SVM classifier under the targeted augmentation strategy. Reliability diagrams revealed substantial calibration drift across the three classes, particularly for Osteopenia and Osteoporosis.

For the Normal class, the model demonstrated under confidence in the low-to-moderate probability range, where observed frequencies were consistently higher than predicted probabilities. Although the model rarely assigned high-confidence predictions for Normal cases, recalibration slightly improved alignment with the ideal calibration curve.

**Figure 14:**
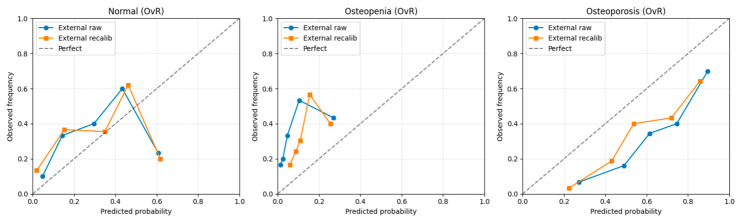
Reliability diagrams comparing raw and recalibrated probabilities for the EfficientNet-B0 + SVM model under external validation. The Normal class exhibited under confidence in the low-to-moderate probability range, whereas the Osteopenia class showed severe probability collapse with predicted probabilities remaining below 0.30 across all bins. The Osteoporosis class demonstrated overconfidence in the raw model, while isotonic recalibration improved probability alignment toward the ideal calibration curve. These findings highlight the presence of calibration drift and class-prior mismatch under external domain shift.

From Figure 14, the most significant finding was observed in the Osteopenia class. Predicted probabilities collapsed into a narrow range below 0.30, indicating that the model almost never assigned high confidence to Osteopenia cases during external testing. Despite low predicted probabilities, the observed Osteopenia frequency remained relatively high, suggesting severe underestimation of this class. Consequently, Osteopenia predictions were frequently overridden by Normal or Osteoporosis during argmax classification, resulting in extremely poor recall performance.

For the Osteoporosis class, the raw model exhibited overconfidence, particularly at high predicted probabilities. However, isotonic recalibration substantially improved probability alignment with the ideal diagonal curve, especially within the moderate probability range. Internal out-of-fold recalibration also demonstrated consistent improvements in calibration quality, confirming that the recalibration pipeline functioned effectively under the internal data distribution.

External confusion matrix analysis further confirmed the presence of prediction collapse toward the Osteoporosis class. A large proportion of Normal cases were incorrectly classified as Osteoporosis, while Osteopenia recall remained nearly absent even after recalibration. Although recalibration improved Normal recall from 14% to 22%, Osteopenia recall remained unchanged at only 2%, indicating that probability recalibration alone was insufficient to recover the suppressed Osteopenia decision region under external domain shift (see Table 9).

### 3.5. Class-Prior Boosting and CV-Unbiased for Minority-Class Recovery

Given that recalibration alone could not recover Osteopenia sensitivity under external validation, an additional class-prior adjustment strategy is recommended. The external results indicate that Osteopenia probabilities remained consistently lower than those of Normal and Osteoporosis across nearly all samples, causing systematic suppression during argmax decision-making.

To address this issue, future work should investigate Class-Prior Boosting or logit-adjusted inference strategies designed to compensate for prior probability mismatch between the balanced training distribution and the real-world external distribution. Such approaches may include posterior probability correction, class-specific threshold optimization, cost-sensitive decision rules, or Bayesian prior adjustment. These methods could help restore the Osteopenia decision region without substantially degrading Osteoporosis sensitivity, thereby improving model performance and calibration under external testing conditions. However, the effectiveness of these strategies should be further evaluated using larger multi-center clinical cohorts before conclusions regarding clinical utility can be drawn.

From Table 10, reliability analysis revealed that the Osteopenia class experienced near-complete probability collapse, with predicted probabilities consistently remaining below 0.30 during external testing. Consequently, Osteopenia samples were systematically overridden by Normal or Osteoporosis predictions during argmax classification, resulting in extremely poor baseline recall (2%). In contrast, the Osteoporosis class exhibited strong overconfidence, while the Normal class showed underconfidence in the low-to-moderate probability range.

Post-hoc isotonic recalibration improved probability reliability and reduced calibration error on the internal distribution, decreasing ECE_top from 0.044 to 0.023 and improving internal accuracy from 0.776 to 0.795. However, recalibration alone was insufficient to recover Osteopenia sensitivity on external data, indicating that the dominant issue was a genuine cross-domain class-prior shift rather than simple probability scaling error.

To address this limitation, class-prior boosting was introduced as a deployment-time adjustment strategy. Experimental analysis identified a favorable operating range at boost coefficients between 4.0 and 5.5, where Osteopenia detection improved substantially without completely degrading performance in the remaining classes. In particular, cross-validated tuning consistently selected a boost coefficient of 5.5 across all five folds, suggesting stable adjustment behavior under the evaluated external testing conditions. However, further evaluation using larger multi-center clinical cohorts is required to determine whether these findings generalize to broader clinical populations.

From Table 11, the CV-unbiased evaluation demonstrated that class-prior adjustment increased overall accuracy and balanced accuracy from 38.0% to 48.0%, while macro F1-score improved from 0.259 to 0.416. Most importantly, Osteopenia recall improved dramatically from 2% to 78%, transforming the model from effectively blind to the intermediate disease stage into a clinically meaningful early-detection screening system. Although Normal recall decreased from 22% to 6%, this trade-off is acceptable within the intended opportunistic screening context, where minimizing missed Osteopenia and Osteoporosis cases is more clinically important than reducing false-positive referrals.

## 4. Discussion

The experimental results demonstrate that the performance of the proposed framework is strongly influenced by both data diversity and cross-domain variability. In this study, data augmentation strategies, particularly the 3× oversampling approach, substantially improved the discriminative capability of the models, as reflected by the increased external AUC values. These findings suggest that minority-class expansion enabled the CNN feature extractors to learn more representative disease-related patterns, especially for Osteopenia and Osteoporosis cases. However, despite the improved ranking performance, threshold-based metrics such as accuracy and F1-score remained substantially lower during external validation, indicating that the primary challenge was not feature learning itself, but rather probability calibration and domain transferability.

The extensive use of augmentation and oversampling should also be interpreted with caution. Although these strategies effectively increased the apparent training sample size and improved minority-class representation, they do not introduce truly independent patient samples and therefore cannot fully replace larger and more diverse datasets. Consequently, there remains a risk that the learned representations may partially reflect cohort-specific characteristics, potentially limiting generalizability under external conditions.

In addition, artificial class balancing modifies the effective class priors observed during training. While this often improves discriminative performance and minority-class detection, it may also distort posterior probability estimates when the model is evaluated on datasets with different class distributions. This phenomenon is consistent with the calibration drift observed during external validation, where relatively strong discrimination performance was accompanied by reduced threshold-dependent metrics. These findings highlight the importance of considering probability calibration alongside conventional classification accuracy when developing AI-based screening systems.

The external validation analysis revealed a clear discrepancy between threshold-independent metrics (AUC) and threshold-dependent metrics (accuracy, precision, recall, and F1-score). While several models achieved relatively high AUC values (0.87–0.89), external classification accuracy remained limited (approximately 42–43%). Importantly, high discrimination performance should not be interpreted as evidence of clinical readiness. Although AUC values remained relatively high under external testing, the substantially reduced classification accuracy indicates that many individual predictions would still be incorrect in practice. Therefore, discrimination alone is insufficient if clinically relevant classification performance remains limited after domain transfer, highlighting the need for calibration-aware and deployment-oriented model evaluation.

This finding indicates that the learned deep features retained discriminatory information under external testing conditions, but the posterior probability distributions became distorted after domain transfer. Such behavior is characteristic of calibration drift in medical imaging AI systems. Despite encouraging discrimination performance, the external validation cohort remained relatively small and originated from a single public dataset. Therefore, the reported results should be interpreted cautiously and should not be considered sufficient evidence for routine clinical deployment. Additional multi-center validation studies involving diverse imaging systems and patient populations are required to further establish generalizability and clinical utility.

Further recalibration analysis using the EfficientNet-B0 + SVM model under the targeted augmentation strategy confirmed the presence of severe class-specific calibration failure. Reliability diagrams demonstrated that the Osteopenia class experienced substantial probability collapse, with predicted probabilities remaining consistently below 0.30 during external testing. Consequently, Osteopenia cases were systematically overridden by Normal or Osteoporosis predictions during argmax classification, resulting in extremely poor recall performance. In contrast, the Osteoporosis class exhibited overconfidence, while the Normal class showed moderate under confidence in low-to-intermediate probability ranges.

Post-hoc isotonic recalibration improved probability reliability on the internal distribution, reducing calibration error and slightly improving external Normal recall. However, recalibration alone was insufficient to recover Osteopenia sensitivity on external data, suggesting that the dominant issue was a true cross-domain class-prior mismatch rather than simple probability scaling error. External confusion matrix analysis further confirmed that the model tended to collapse toward Osteoporosis predictions, leading to a high false-positive rate for severe disease categories.

To address this limitation, class-prior boosting and CV-unbiased threshold optimization were introduced as deployment-oriented correction strategies. The results demonstrated a substantial improvement in minority-class recovery, particularly for Osteopenia detection. Cross-validated post-hoc adjustment consistently selected the same boost coefficient across all external folds, indicating that the observed correction suggested a consistent adjustment pattern under the evaluated external testing conditions. Most importantly, Osteopenia recall improved markedly from near-complete failure at baseline to substantially higher detection rates after adjustment, accompanied by improvements in balanced accuracy and macro F1-score.

The clinical implications of this improvement should be interpreted carefully. While class-prior boosting substantially increased Osteopenia detection, the gain was accompanied by reductions in recall for the Normal and Osteoporosis classes. Therefore, the observed improvement should not be viewed as universally beneficial but rather as a trade-off between competing clinical objectives. In particular, although increased false-positive referrals may be acceptable in an opportunistic screening setting, missed Osteoporosis cases remain clinically important because delayed identification may postpone further assessment and treatment in patients at elevated fracture risk. Consequently, the optimal boosting strategy should be selected according to the intended clinical use case and the relative importance assigned to sensitivity and specificity across the three diagnostic categories.

Although Normal and Osteoporosis recall decreased after aggressive minority-class boosting, this trade-off may be acceptable within the intended opportunistic screening context. In future deployment settings, the proposed framework is not intended to replace DXA-based diagnosis, but rather to function as a pre-screening or referral-support system for identifying patients who may require further osteoporosis assessment. Under such scenarios, improving sensitivity for patients at risk of reduced bone density must be balanced against the need to maintain adequate detection of established Osteoporosis cases.

The results also emphasize the importance of dataset diversity and multi-center validation. Variations in X-ray acquisition systems, detector characteristics, image contrast, noise profiles, and patient demographics substantially influence feature distributions and probability calibration across institutions. Therefore, future research should focus on collecting larger multi-center datasets, performing device-aware external evaluation and formal multi-center clinical validation, and integrating advanced domain-adaptive calibration or class-prior correction methods into the deployment pipeline.

Direct comparison with previously published osteoporosis classification studies should be interpreted cautiously, because substantial differences exist in dataset composition, imaging protocols, patient populations, class definitions, and evaluation methodologies. While several studies have reported higher classification accuracies, many were conducted using internal datasets without independent external validation. In contrast, the present study specifically investigated external generalization, calibration drift, and deployment-oriented correction strategies, including isotonic recalibration and class-prior boosting. These analyses provide additional insights into the challenges of translating AI-based osteoporosis screening systems from independent external testing datasets to broader clinical environments.

From a clinical perspective, opportunistic osteoporosis assessment from routine knee radiographs may provide valuable information beyond the primary evaluation of knee pathology. Patients undergoing assessment for osteoarthritis, knee pain, or consideration for arthroplasty routinely receive knee radiography as part of standard clinical care. Automated identification of Osteopenia or Osteoporosis from these existing images may support earlier referral for DXA assessment and assist clinicians in identifying patients who may benefit from further osteoporosis evaluation. Nevertheless, the present framework should be regarded as a proof-of-concept approach. Although the findings suggest potential clinical utility, further validation using larger, multi-center, and prospectively collected cohorts is required before routine clinical implementation can be considered.

An additional limitation of this study is the relatively small clinical dataset, which consisted of 116 patients from a single institution. Consequently, the findings should be interpreted as preliminary evidence of feasibility rather than definitive evidence of clinical effectiveness. Although transfer learning, data augmentation, and class balancing were employed to improve model stability and generalization, the available data may not fully capture the variability encountered in broader clinical populations. This limitation is reflected in the observed performance gap between internal and external validation, highlighting the challenges of generalization across different imaging domains. Future studies should therefore include larger multi-center cohorts and prospective validation to further assess model robustness, generalizability, and clinical applicability.

Overall, the proposed framework demonstrates that pretrained CNN-based deep feature extraction, combined with machine learning classifiers, can capture osteoporosis-related imaging patterns from knee X-ray images. More importantly, this study highlights that future clinical translation of AI-based osteoporosis screening systems requires not only strong feature extraction capability, but also explicit handling of calibration drift, class-prior mismatch, and cross-domain variability through adaptive recalibration and deployment-aware threshold optimization strategies.

## 5. Conclusions

This study presents a multi-stage framework for osteoporosis classification from knee X-ray images by integrating deep feature extraction with machine learning classifiers. The experimental results demonstrate that pretrained CNN models, particularly EfficientNetB0 and DenseNet121, are capable of extracting informative and discriminative features associated with osteoporosis status. Among the evaluated classifiers, Neural Network, Efficient Linear, and SVM-based models exhibited stable and reliable performance when combined with deep CNN representations. A key methodological contribution of this work is the integration of complementary deep feature representations, machine-learning-based classification, external validation, and post-hoc calibration analysis within a single translational framework. Unlike conventional single-model approaches, the proposed architecture explicitly addresses both predictive discrimination and deployment-related challenges associated with cross-domain generalization.

The findings further emphasize the importance of data preparation strategies, including targeted augmentation, oversampling, and class balancing. These approaches improved feature separability and minority-class representation, leading to enhanced discriminative performance, particularly in terms of AUC. Nevertheless, the results also indicate that model performance remains strongly influenced by dataset size, diversity, and inter-domain variability, suggesting that larger and more representative datasets are essential for improving model stability and generalizability.

A major contribution of this study is the identification of calibration drift and class-prior mismatch during external validation. Although several models maintained relatively high AUC values under external testing, threshold-dependent metrics such as accuracy and F1-score decreased substantially. This finding underscores that strong discrimination alone is insufficient to support clinical deployment when threshold-based classification performance remains limited under external conditions. Reliability analysis revealed that the primary limitation was not the loss of learned feature representations, but rather distorted posterior probability distributions across domains, especially for the intermediate Osteopenia class.

To address this issue, post-hoc recalibration and class-prior boosting strategies were investigated. The proposed post-hoc adjustment strategy improved minority-class recovery under the evaluated external validation setting, particularly for Osteopenia detection, and enhanced balanced accuracy and macro F1-score during external validation. These findings suggest that adaptive recalibration and threshold optimization may be important components for future clinical translation of AI-based osteoporosis screening systems.

From a clinical perspective, the proposed framework demonstrates the potential of opportunistic osteoporosis assessment using routine knee radiographs. The proposed framework is intended as an opportunistic screening and decision-support tool that can be integrated into routine radiographic workflows. Patients identified as being at elevated risk of osteopenia or osteoporosis from existing knee radiographs may be prioritized for confirmatory DXA assessment and further clinical evaluation. In this manner, the framework has the potential to support early risk identification without replacing established diagnostic procedures.

Overall, this study provides proof-of-concept evidence that a multi-stage CNN and machine learning framework can extract informative osteoporosis-related patterns from routine knee radiographs. The findings further demonstrate the importance of addressing calibration drift and class-prior mismatch when transferring AI models across imaging domains. Nevertheless, the study is limited by its single-center internal dataset and relatively small external validation cohort derived from an independent public dataset. Additional multi-center, prospective validation studies and further calibration research are required before routine clinical implementation can be considered. Despite these limitations, the present findings demonstrate that routine knee radiographs contain clinically relevant information for osteoporosis risk stratification and highlight the importance of combining external validation, calibration analysis, and adaptive threshold optimization when developing clinically transferable AI systems. These principles may serve as a useful foundation for future AI-assisted musculoskeletal screening applications.

## Figures and Tables

**Table 1:** Dataset distribution across processing stages.

Stage	Normal	Osteopenia	Osteoporosis	Total
Original Dataset	119	90	113	322
Training Set (80%)	95	72	90	257
Test Set (20%)	24	18	23	65
After Targeted Augmentation	95	216	270	581
After All 3× Augmentation (Train Only)	285	216	270	771
After 3× + Class Balancing (Final Train Set)	285	285	285	855

**Table 2:** Comparison of deep learning models for feature extraction.

Model	Input Size	Feature Extraction Layer	Feature Dimension	Core Block	Feature Extraction Characteristics	Strengths	Limitations
ResNet18	224 × 224 × 3	pool5	512	Residual Block	Utilizing residual connections to enable stable deep feature learning and mitigate vanishing gradients	Lightweight, fast computation, compact feature representation	Limited feature richness compared to deeper models
EfficientNetB0	224 × 224 × 3	global_average_pooling2d_1	1280	MBConv + SE	Employing compound scaling with mobile inverted bottleneck convolution and squeeze-and-excitation for efficient feature extraction	High efficiency, balanced performance, strong representation capability	More complex architecture, less interpretable
DenseNet-121	224 × 224 × 3	avg_pool	1920	Dense Block	Dense connectivity promotes feature reuse and strengthens gradient propagation, producing highly informative features.	Rich feature representation, excellent information flow	High computational cost, large feature dimension

**Table 3:** Optimized hyperparameters and search ranges for machine learning classifiers in MATLAB Classification Learner.

Classifier	Optimized Hyperparameters	Final Selected Values	Hyperparameter Search Range	Standardization	Optimization Method
Tree	Maximum Number of Splits, Split Criterion	Maximum Splits = 27; Criterion = Maximum Deviance Reduction	Maximum Splits: 1–854; Criterion: Gini, Twoing, Maximum Deviance Reduction	N/A	Bayesian Optimization
SVM	Kernel Function, Box Constraint, Multiclass Coding	Kernel = Quadratic; Box Constraint = 0.0010306; Coding = One-vs-All	Kernel: Gaussian, Linear, Quadratic, Cubic; Box Constraint: 0.001–1000; Coding: One-vs-All, One-vs-One	No	Bayesian Optimization
Naive Bayes	Distribution Name, Kernel Type, Standardization	Distribution = Gaussian; Kernel = Epanechnikov	Distribution: Gaussian, Kernel; Kernel: Gaussian, Box, Epanechnikov, Triangle	No	Bayesian Optimization
KNN	Number of Neighbors, Distance Metric, Distance Weight	Neighbors = 1; Distance = Spearman; Weight = Inverse	Neighbors: 1–428; Distance: Cityblock, Chebyshev, Correlation, Cosine, Euclidean, Spearman; Weight: Equal, Inverse, Squared Inverse	Yes	Bayesian Optimization
Neural Network	Number of Layers, Activation Function, Lambda, Layer Sizes	Layers = 2; Activation = ReLU; Lambda = 1.5367 × 10^−5^; Layer1 = 285; Layer2 = 17	Layers: 1–3; Activation: ReLU, Tanh, Sigmoid, None; Lambda: 1.1696 × 10^−8^–116.9591; Layer Sizes: 1–300	Yes	Bayesian Optimization
Efficient Linear	Learner Type, Regularization, Lambda, Multiclass Coding	Learner = Logistic Regression; Regularization = Ridge; Lambda = 1.4763 × 10^−5^; Coding = One-vs-One	Learner: Logistic Regression, SVM; Regularization: Ridge, Lasso; Lambda: 1.1696 × 10^−8^–116.9591; Coding: One-vs-All, One-vs-One	Auto	Bayesian Optimization

**Table 4:** Performance comparison of optimizable machine learning classifiers using deep features extracted from ResNet18, EfficientNet-B0, and DenseNet-121 under the minority class expansion (3× augmentation) strategy. Results are reported in terms of accuracy, precision, recall, F1-score, training time, and AUC (Area Under the Receiver Operating Characteristic Curve) for both validation and test sets.

CNN Extract Feature	Classification Model	Training Time	Accuracy		Precision		Recall		F1-Score		AUC
			Valid	Test	Valid	Test	Valid	Test	Valid	Test	
ResNet18	Tree	66.02	51.81	35.38	46.05	36.71	45.25	34.18	44.99	32.61	0.5486
	SVM	116.62	78.31	50.77	78.52	57.39	74.65	51.37	76.17	50.28	0.7251
	Naive Bayes	845.41	53.18	50.77	49.91	55.28	50.43	49.64	49.81	49.16	0.6763
	KNN	48.08	70.91	41.54	69.94	48.37	65.37	42.33	66.59	41.93	0.5710
	Neural Network	587.83	72.46	58.46	71.21	64.85	70.12	59.18	70.62	58.93	0.7277
	Efficient Linear	104.56	67.81	61.54	68.14	67.13	66.12	61.96	67.00	62.17	0.7865
EfficientNetB0	Tree	188.17	56.80	47.69	58.04	56.09	50.14	47.38	51.59	45.18	0.6766
	SVM	378.69	79.52	70.77	81.17	74.36	76.81	68.40	78.51	68.30	0.8409
	Naive Bayes	994.32	62.82	67.69	61.76	68.67	62.79	64.63	61.86	63.35	0.7593
	KNN	317.40	69.19	53.85	68.63	57.47	60.83	54.09	61.83	53.85	0.6574
	Neural Network	1070.51	77.11	73.85	77.53	77.17	75.26	72.10	76.25	72.47	0.8398
	Efficient Linear	496.91	77.80	72.31	78.21	72.98	75.39	69.73	76.57	69.67	0.8887
DenseNet-121	Tree	227.54	56.80	41.54	56.18	41.79	52.95	40.30	54.04	40.82	0.6088
	SVM	393.54	82.79	61.54	84.19	63.75	81.22	60.23	82.48	60.97	0.8530
	Naive Bayes	3184.58	60.41	56.92	58.75	53.63	59.98	54.49	59.27	53.47	0.6889
	KNN	950.23	76.94	50.77	76.75	53.27	71.11	49.86	72.80	50.69	0.6540
	Neural Network	2415.21	84.51	67.69	84.81	66.45	84.37	65.78	84.56	66.02	0.8557
	Efficient Linear	660.84	78.31	70.77	80.16	71.53	77.52	69.61	78.68	69.95	0.8409

**Table 5:** Class-wise sensitivity, specificity, and positive predictive value (PPV) for the best-performing CNN–classifier combinations on the internal test set.

Model	Class	Sensitivity (%)	Specificity (%)	PPV (%)
ResNet18 + Efficient Linear	Normal	58.33	97.56	93.33
	Osteopenia	66.67	76.60	41.38
	Osteoporosis	60.87	88.10	66.67
EfficientNetB0 + SVM	Normal	75.00	97.56	94.74
	Osteopenia	38.89	92.55	70.00
	Osteoporosis	91.30	65.12	58.33
EfficientNetB0 + Neural Network	Normal	75.00	97.56	94.74
	Osteopenia	50.00	92.55	75.00
	Osteoporosis	91.30	72.09	61.76
EfficientNetB0 + Efficient Linear	Normal	83.33	97.56	95.24
	Osteopenia	38.89	89.36	58.33
	Osteoporosis	86.96	74.42	60.61
DenseNet121 + Efficient Linear	Normal	75.00	97.56	94.74
	Osteopenia	55.56	85.11	55.56
	Osteoporosis	78.26	79.07	64.29

**Table 6:** Performance comparison of optimizable machine learning classifiers using deep features extracted from ResNet18, EfficientNet-B0, and DenseNet-121 under the full dataset expansion and class balancing (3× oversampling) strategy. Results are reported in terms of accuracy, precision, recall, F1-score, training time, and AUC for both validation and test sets.

CNN Extract Feature	Classification Model	Training Time	Accuracy		Precision		Recall		F1-Score		AUC
			Valid	Test	Valid	Test	Valid	Test	Valid	Test	
ResNet18	Tree	124.29	50.88	49.23	52.65	52.01	50.88	49.09	50.85	49.17	0.6423
	SVM	437.58	76.84	55.38	76.70	51.72	76.84	52.76	76.76	51.82	0.7754
	Naive Bayes	437.10	54.85	53.85	54.88	44.60	54.85	50.10	54.77	46.03	0.7049
	KNN	156.04	76.49	50.77	76.78	53.55	76.49	50.26	76.55	50.93	0.6311
	Neural Network	1131.68	75.91	66.15	75.73	61.40	75.91	63.00	75.80	61.48	0.7893
	Efficient Linear	152.69	73.45	63.08	73.27	53.80	73.45	58.84	73.35	55.58	0.8208
EfficientNetB0	Tree	856.79	55.79	55.38	56.27	54.89	55.79	53.16	55.63	52.64	0.7666
	SVM	850.41	87.72	67.69	87.70	66.83	87.72	65.44	87.71	65.37	0.8707
	Naive Bayes	1618.84	63.51	61.54	64.19	63.16	63.51	58.55	63.72	57.38	0.7426
	KNN	154.09	82.11	61.54	82.73	60.09	82.11	59.76	82.26	59.70	0.7029
	Neural Network	1089.81	86.78	67.69	86.90	65.88	86.78	65.32	86.83	64.78	0.8735
	Efficient Linear	191.22	83.16	75.38	83.21	76.21	83.16	72.10	83.18	70.77	0.8880
DenseNet-121	Tree	368.44	58.25	55.38	58.34	55.85	58.25	53.62	58.28	54.22	0.6795
	SVM	1743.66	88.89	64.62	88.94	60.41	88.89	61.61	88.78	60.23	0.8470
	Naive Bayes	4185.09	61.99	52.31	61.63	47.37	61.99	49.34	61.72	47.14	0.6671
	KNN	275.49	86.90	61.54	86.87	60.05	86.90	59.70	86.86	59.51	0.7016
	Neural Network	2265.97	86.55	75.38	86.44	72.79	86.55	72.85	86.47	72.45	0.9010
	Efficient Linear	310.42	82.11	73.85	82.18	72.47	82.11	72.32	82.11	72.37	0.8929

**Table 7:** Class-wise sensitivity, specificity, and positive predictive value (PPV) of CNN–classifier models trained using the 3× oversampling and class-balancing strategy.

Model	Class	Sensitivity (%)	Specificity (%)	PPV (%)
ResNet18 + Neural Network	Normal	91.67	85.37	75.86
	Osteopenia	27.78	85.11	41.67
	Osteoporosis	69.57	79.07	66.67
EfficientNetB0 + Efficient Linear	Normal	91.67	92.68	88.00
	Osteopenia	33.33	93.62	75.00
	Osteoporosis	91.30	72.09	65.63
DenseNet121 + Neural Network	Normal	95.83	95.12	92.00
	Osteopenia	44.44	89.36	57.14
	Osteoporosis	78.26	81.40	69.23

**Table 8:** External validation results of different CNN-based feature extraction models combined with machine learning classifiers. Performance metrics including accuracy, precision, recall, F1-score, and AUC are reported for both validation and test sets to evaluate model generalization on an independent knee X-ray dataset.

CNN Extract Feature	Classification Model	Accuracy		Precision		Recall		F1-Score		AUC
		Valid	Test	Valid	Test	Valid	Test	Valid	Test	
(EfficientNetB0) Targeted Augmentation	SVM	78.31	44.00	78.52	44.66	74.65	44.00	76.17	38.79	0.7251
	Neural Network	72.46	38.67	71.21	36.76	70.12	38.67	70.62	35.84	0.7277
	Efficient Linear	67.81	43.33	68.14	42.03	66.12	43.33	67.00	39.90	0.7865
(EfficientNetB0) 3× + Oversampling	Neural Network	86.78	42.00	86.90	41.32	86.78	42.00	86.83	40.16	0.8735
	Efficient Linear	83.16	39.33	83.21	37.71	83.16	39.33	83.18	37.35	0.8880
(DenseNet-121) 3× + Oversampling	Neural Network	86.78	43.33	86.90	37.29	86.78	43.33	86.83	38.26	0.8735
	Efficient Linear	83.16	31.33	83.21	28.92	83.16	31.33	83.18	29.43	0.8880

**Table 9:** Internal calibration performance and external per-class recall after isotonic recalibration using EfficientNet-B0 + SVM under the targeted augmentation strategy.

Metric	Raw Model	Recalibrated Model
Internal OOF Accuracy	0.776	0.795
ECE_top	0.044	0.023
ECE_cw	0.047	0.017
Brier Score	0.327	0.307
Class	Raw Recall (%)	Recalibrated Recall (%)
Normal	14	22
Osteopenia	2	2
Osteoporosis	94	90

**Table 10:** Internal calibration performance and external per-class recall before and after isotonic recalibration for the EfficientNet-B0 + SVM model under the targeted augmentation strategy.

Boost	Accuracy	Normal	Osteopenia	Osteoporosis	Balanced Acc
1.0 (baseline)	0.380	22%	2%	90%	38.0%
4.0	0.447	20%	28%	86%	44.7%
5.0	0.453	16%	60%	60%	45.3%
7.0	0.453	4%	84%	48%	45.3%
10.0	0.367	0%	96%	14%	36.7%

**Table 11:** Performance comparison of class-prior boosting strategies on external validation data, including overall accuracy, balanced accuracy, macro F1-score, and per-class recall for Normal, Osteopenia, and Osteoporosis categories.

Performance	Baseline (Boost = 1)	CV-Tuned (Boost = 5.5)	Δ
Accuracy	0.380	0.480	+10.0 pp
Balanced Accuracy	0.380	0.480	+10.0 pp
Macro F1	0.259	0.416	+15.7 pp
Normal recall	22%	6%	−16 pp
Osteopenia recall	2%	78%	+76 pp
Osteoporosis recall	90%	60%	−30 pp

## Data Availability

Restrictions apply to the availability of these data. The internal knee radiograph dataset was obtained from the University of Phayao Hospital and is not publicly available due to ethical and privacy restrictions but may be available from the corresponding author upon reasonable request and with permission from the University of Phayao Hospital. The external dataset used in this study was obtained from Kaggle and is available at https://www.kaggle.com/datasets/mohamedgobara/multi-class-knee-osteoporosis-x-ray-dataset (accessed 1 November 2025) with the permission of Kaggle.
